# Co-infection With Dengue Virus and Extensively Drug-Resistant Salmonella typhi

**DOI:** 10.7759/cureus.19653

**Published:** 2021-11-17

**Authors:** Said Amin, Mohammad Noor, Fawad Rahim, Muhammad Yasir Khan, Raza Ullah

**Affiliations:** 1 Internal Medicine, Khyber Girls Medical College, Peshawar, PAK; 2 Internal Medicine, Hayatabad Medical Complex Peshawar, Peshawar, PAK; 3 Critical Care Medicine, Hayatabad Medical Complex Peshawar, Peshawar, PAK

**Keywords:** drug resistance, coexistent disease, dengue fever, salmonella typhi, enteric fever

## Abstract

In South Asia, infectious diseases are associated with significant morbidity and mortality. Malaria, typhoid, and dengue are the most common infectious diseases, and patients may be co-infected with these diseases, resulting in diagnostic and treatment dilemmas.

Dengue is caused by the *Arboviridae *family of viruses and is transmitted by *Aedes aegypti*. Dengue virus has four serotypes and the symptoms of dengue fever mimic those of other infectious diseases such as malaria, chikungunya, Zika virus disease, influenza A, enteric fever, and coronavirus disease 2019, which are also prevalent in areas of frequent dengue fever outbreak. Dengue fever is characterized by fever, severe myalgia, retro-orbital pain, skin rashes, and bone pain, which is why dengue fever is also referred to as breakbone fever. Patients with secondary dengue virus infection more commonly present with abdominal pain when compared with those with primary dengue virus infection.

*Salmonella typhi *causes enteric fever, which is a human infection with no animal reservoir, but the symptoms of enteric fever closely resemble those of dengue fever. Concurrent infections with multiple organisms are especially difficult to diagnose.

In this report, we present the case of a 15-year-old boy from Peshawar, Pakistan, who was co-infected with the dengue virus and extensively drug-resistant *Salmonella typhi*. To the best of our knowledge, this is the first reported case of co-infection with dengue virus and extensively drug-resistant Salmonella typhi.

## Introduction

Dengue fever is a viral disease. It is transmitted through the bite of the mosquito, *Aedes aegypti*. It is commonly observed during the monsoon season in tropical regions and less commonly in subtropical regions. Dengue fever is caused by one of four serotypes of the dengue virus: DEN1, DEN2, DEN3, and DEN4. The disease is characterized by fever, rash, myalgia, anorexia, joint and bone pain, and headache [1]. In most patients, it follows a mild course with no need for inpatient care. Some patients may develop complications such as hemorrhage and shock, which can be fatal. Thrombocytopenia, low white blood cell count, and elevated levels of liver enzymes, particularly alanine transaminase (ALT), are common in dengue fever. Thrombocytopenia can be mild with no need for specific treatment except close observation; however, it can sometimes be severe and even fatal. Patients with dengue fever may also have gastrointestinal and neurological symptoms [2].

Infectious diseases are common in Pakistan. The first reported outbreak of dengue fever in Pakistan was in 1994. According to the National Institute of Health Islamabad, Pakistan, there were 22,938 cases of dengue fever in 2017, 3,200 cases in 2018, 24,547 cases in 2019, and 3,442 cases in 2020 [3]. In Pakistan, the most prevalent circulating serotype is DEN2, and there are a few reported cases involving DEN3. The incidence of dengue fever in Pakistan in 2021 is reported to be increasing, especially in Peshawar, Lahore, and the twin cities of Rawalpindi and Islamabad [4].

*Salmonella typhi* infection presents with fever, anorexia, body aches, and gastrointestinal symptoms such as abdominal pain and diarrhea. It may be complicated by gastrointestinal bleeding and neurological symptoms including encephalopathy. The characteristic laboratory abnormalities in patients with enteric fever are leukopenia and elevated ALT levels [5]. The initial presentation and some complications of dengue fever and enteric fever are closely similar. In a 2016 report to the World Health Organization (WHO), it was stated 5,274 out of 8,188 (64.4%) *Salmonella typhi *cultured in the Sindh province of Pakistan are extensively drug-resistant (XDR). Karachi accounted for 69% of the cases of XDR *Salmonella typhi*. Hyderabad accounted for 27% of the cases, and other districts of Sindh province accounted for 4% of the cases [6]. The Centers for Disease Control warned that travelers to Pakistan are at risk of infection with the XDR *Salmonella typhi *strain. It was also reported that planned family visits are associated with a greater risk of XDR *Salmonella typhi *infection than tourist trips or business trips. Additionally, XDR *Salmonella typhi *infection was reported in patients who traveled from Pakistan to the United States, the United Kingdom, and Canada [7]. Although, co-infection with dengue virus and *Salmonella typhi *has been previously reported, but as far as we know, this is the first report of co-infection with dengue virus and XDR *Salmonella typhi*.

## Case presentation

A 15-year-old boy presented to the emergency medical unit with intermittent high-grade fever of a duration of five days, generalized body aches, abdominal pain, loose watery stools with no blood or mucus, and dry cough. Physical examination revealed a pulse of 92 beats/minute, blood pressure of 100/70 mmHg in the right arm in a supine position, and a body temperature of 39 degrees Celsius. The rest of the physical examination was normal except for diffuse periumbilical tenderness. His full blood count revealed hemoglobin level of 12 gram/deciliter, total leukocyte count of 3,100/mcL (with 70% neutrophils, 25% lymphocytes, 2% basophils, and 3% monocytes), and platelet count of 28,000/mcL. His hematocrit was 31.9%, and he was positive for dengue nonstructural antigen 1.

He was started on WHO-approved treatment protocol for dengue fever. On day 8 after symptom onset, his platelet count increased to 54,000/mcL, but he remained febrile (body temperature: 40 degrees Celsius) and toxic, with abdominal pain and loose watery stools. He was reviewed for other causes of persistent fever, and baseline investigations were repeated. His blood and stool samples were sent for culture. The results of the serology tests for hepatitis viruses A, B, C, and E, repeated thick and thin smears for malarial parasites, and human immunodeficiency virus antibodies were negative. He underwent contrast-enhanced computed tomography of the abdomen and pelvis, which was unremarkable. Table 1 shows the results of serial full blood counts and liver function tests. He was started on meropenem empirically. The preliminary report of the blood culture indicated a growth of gram-negative rods. The final report revealed an XDR *Salmonella typhi* strain. Table 2 shows the sensitivity report. Considering his culture report, meropenem was continued, and the patient’s condition improved over time. He was discharged home after 20 days of admission. A week later, he was reviewed again. He remained afebrile. 

**Table 1:** Serial laboratory investigations

Variable	Reference range	Day 1	Day 3	Day 5	Day 7	Day 9	Day 11	Day 15
Hemoglobin level (g/dL)	13.5 – 17.5	11.4	11.0	9.58	8.57	9.37	7.7	9.5
Platelet count (×10^3^/mcL)	150 – 450	28	48.2	52.8	50.4	45.5	54	80
Total leukocyte count (x10^3^/mcL)	7 – 11	9.5	9.7	10.2	10.4	9.9	9.8	10.1
Alanine transaminase level (IU/L)	10 – 45	195	235	144	182	191	214	110

**Table 2:** Blood culture and sensitivity report

Drug	Sensitivity
Ceftriaxone	Resistant
Ampicillin	Resistant
Cefixime	Resistant
Chloramphenicol	Resistant
Co-trimoxazole	Resistant
Ciprofloxacin	Resistant
Azithromycin	Sensitive
Imipenem	Sensitive
Meropenem	Sensitive

## Discussion

The emergence and coexistence of infections with a potential for global spread are a significant health concern, especially in countries with low sanitation levels and limited health resources [8]. Malaria, typhoid, and dengue fever are the most common acute febrile illnesses in Pakistan. They mostly have similar seasonal patterns and occasionally coexist, resulting in a diagnostic dilemma for physicians.

In this report, we present the case of a young boy with co-infection of dengue fever and XDR *Salmonella typhi*. Co-infections with the dengue virus and *Salmonella typhi *have been reported in case reports and cross-sectional studies. All the cross-sectional studies relied on serological diagnosis of typhoid fever using the Widal test, rather than a culture, which is a gold standard test for typhoid fever diagnosis [9-11] By contrast, blood cultures were used to diagnose *Salmonella typhi *infection in case reports of co-infection with dengue virus in children and adults. A case of dengue and typhoid co-infection in India was reported, but results of a sensitivity test were not provided [12]. In two patients with dengue fever, culture-proven *Salmonella typhi *was found to be sensitive to fluoroquinolones [13,14], and in four other patients, *Salmonella typhi *was found to be sensitive to cephalosporins [14-17]. The clinical condition of all the patients improved after treatment with fluoroquinolone and/or oral or parenteral cephalosporin. To the best of our knowledge, there have been no reports of co-infection with an XDR *Salmonella typhi *strain and dengue virus.

The presence of two potentially lethal diseases in our locality is a serious health concern, especially when all conditions that favor disease spread are present. It is clinically challenging to diagnose typhoid fever in a patient with acute fever when an epidemic of dengue fever is ongoing, with hundreds of patients presenting to a healthcare facility on a daily basis [18]. The epidemic significantly increases the demand for diagnostic facilities in countries with limited resources such as Pakistan. The diagnostic challenge is worsened by the clinical resemblance of the concurrence of two different pathogens. Body aches, myalgias, altered bowel patterns, and sore throat are common in both dengue fever and typhoid fever. Similar laboratory findings and physical signs in typhoid fever and dengue fever, including rash, mild splenomegaly, relative bradycardia, and neutropenia make differentiation almost impossible [16]. Other febrile illnesses should be considered, especially when the clinical course of a patient differs from the natural disease course or when a patient is unresponsive to standard treatment.

## Conclusions

This case emphasizes the importance of considering other possibilities when the clinical course of patients with dengue fever differs from the natural course of the disease. Differences in the clinical course may include symptoms such as persistent high-grade fever. All such patients should be evaluated as per protocol for acute febrile illness, including sending blood samples for culture before starting empirical antibiotic therapy. In countries like Pakistan where XDR strains of *Salmonella typhi* have been reported, empirical antibiotic therapy should include agents that cover XDR *Salmonella typhi*, pending blood culture results.
